# Comprehensive Characterization of *BrSULTRs* Family and Their Expression Profiles Under Salt and Low-Temperature Stresses

**DOI:** 10.3390/genes17040394

**Published:** 2026-03-30

**Authors:** Shangjia Liu, Bingxue Han, Zekun Hu, Xiaojia Yin, Xiaoyan Wang, Fengchao Cui

**Affiliations:** 1 The College of Agriculture, Qingdao Hengxing University of Science and Technology, Qingdao 266100, China; 2 State Key Laboratory of Crop Biology, Shandong Agricultural University, Tai’an 271018, China

**Keywords:** *Brassica rapa*, sulfate transporter, abiotic stress, gene expression profile

## Abstract

Background: *Sulfate transporters* (*SULTRs*) are integral membrane proteins responsible for sulfate uptake, translocation, and plant adaptation to abiotic stresses. However, knowledge regarding the *SULTR* gene family in the economically important crop, *Brassica rapa* (Chinese cabbage), limited. The aim of this study is to conduct a genome-wide identification and functional characterization of *BrSULTR* genes and to explore their potential functions under abiotic stress. Methods: We identified 19 *BrSULTR* genes in the *B. rapa* genome by performing homology searches with *Arabidopsis thaliana SULTR* sequences as queries. Subsequent bioinformatics analysis included phylogenetic classification, chromosomal localization, gene structure, conserved motif dissection, *cis*-regulatory element prediction, and protein–protein interaction (PPI) network analysis. Tissue-specific expression profiles of *BrSULTRs* were assessed using publicly available transcriptome data. Furthermore, their expression dynamics under salt (150 mM NaCl) and low-temperature (4 °C) stress were investigated by integrating transcriptomic, proteomic, and qRT-PCR data. Results: The 19 identified *BrSULTR* members were phylogenetically categorized into four subfamilies and were mapped unevenly across seven chromosomes. Promoter analysis identified an array of *cis*-regulatory elements associated with development, hormone response, and stress response. Expression profiles revealed distinct tissue-specific patterns in roots, stems, leaves, flowers, and siliques. Under salt stress, *BrSULTR13* was significantly upregulated, while *BrSULTR9* and *BrSULTR11* were significantly suppressed under low-temperature stress. PPI network projection indicated that the *Arabidopsis* homologs of *BrSULTR5* may physically interact with stress-regulating enzymes such as APS and APR. Conclusions: Our work presents a comprehensive genomic and functional overview of the *BrSULTR* gene family in *B. rapa.* The results underscore the potential functions of *BrSULTRs*, highlighting their involvement in sulfate transport and abiotic stress responses. These insights establish valuable insights and a foundation for further research aiming at improving stress tolerance in *B. rapa* through the manipulation of sulfur metabolism pathways.

## 1. Introduction

Sulfur, as a fundamental macronutrient for plant growth and development, plays an essential role in the synthesis of sulfur-containing organic compounds, including cysteine, methionine, and other essential amino acids [1,2]. It exerts various regulatory functions in plant physiology: (1) as an indispensable component of protein synthesis, it directly influences enzymatic activity and metabolic pathway efficiency; (2) it participates in glutathione biosynthesis and other antioxidant production to maintain cellular redox homeostasis; (3) it plays a pivotal regulatory role in the plant stress response. Sulfur contributes to abiotic stress adaptation through multiple interconnected pathways, forming a comprehensive defense network and enhancing plant stress resistance. Glutathione (GSH) acts as a major cellular antioxidant. Firstly, as a structural component of cysteine, sulfur serves as the foundational precursor for the synthesis of GSH, which directly scavenges reactive oxygen species (ROS) that accumulate under various abiotic stresses such as salinity and low temperature [3]. Secondly, sulfur is a component of the biosynthesis of phytochelatins, which are cysteine-rich peptides synthesized by glutathione enzyme. These peptides form stable complexes by chelating toxic ions such as cadmium, arsenic, and lead, and are isolated in vacuoles, playing a crucial role in heavy metal detoxification. This process not only alleviates metal toxicity but also reduces oxidative stress caused by heavy metals, directly linking sulfur metabolism with metal stress tolerance. Thirdly, sulfur is a key component of iron–sulfur clusters and various sulfur-containing cofactors, which is crucial for the activity and stability of major antioxidant enzymes. For example, the dismutation activity of superoxide dismutase (SOD) depends on sulfur-containing metal cofactors, while catalase and peroxidases typically require sulfur-mediated structural integrity to function [4]. Therefore, maintaining sulfur homeostasis is not only a metabolic requirement, but also a strategic determinant for plants to maintain redox balance, perform detoxification processes, and establish effective physiological defense capabilities under adverse environmental conditions [5,6].

Sulfate (SO_4_^2−^) absorption from the soil is a tightly controlled process mediated by proton (H^+^)-dependent *sulfate transporters* (*SULTRs*), which belong to multiple gene families [7,8]. These integral membrane proteins typically feature 12 transmembrane domains and a C-terminal sulfate transporter and anti-sigma (STAS) domain. They are strategically localized in the plasma membrane to facilitate both sulfate uptake and long-distance transport. The STAS domain fulfills a dual role in maintaining transporter activity and mediating protein-protein interactions, serving as a molecular signature for identifying conserved functional domains in *SULTR* genes [9,10,11,12]. Evolutionary pressure has driven plants to develop a sophisticated sulfate transport system comprising three specialized transporter types: plasma membrane sulfate transporters, thiosulfate transporters, and ATP-dependent sulfate transporters [12]. This tripartite system represents a remarkable evolutionary adaptation to diverse environmental conditions, enabling plants to maintain optimal sulfur acquisition across varying soil compositions and stress scenarios.

*Sulfate transporters* (*SULTRs*) have been characterized in a range of plant species, including *A. thaliana* [12], *Oryza sativa* [13], *Triticum aestivum* [5], *Brassica oleracea* [8], and *Glycine max* [14]. In *A. thaliana*, 12 *SULTRs* have been classified into four subfamilies based on sequence homology [7,15]. Group I consists of high-affinity SULTR proteins. The root-localized high-affinity transporters *AtSULTR1;1* and *AtSULTR1;2* are key regulators of sulfate acquisition [15,16]. *AtSULTR1;3*, which is expressed in the phloem, is also involved in sulfate transport [17]. Group II comprises low-affinity *SULTR* genes, *AtSULTR2;1* and *AtSULTR2;2*, which are predominantly found in root and leaf vascular tissues, where they mediate long-distance sulfate translocation [18]. Specifically, *AtSULTR2;1* facilitates sulfate delivery to siliques [19], while the promoter region of *AtSULTR2;2* contains *cis*-elements essential for expression in bundle sheath and veins-a feature conserved among Brassicaceae *SULTR2;2* homologs [20]. Group III represents the largest *SULTR* subfamily. In *Arabidopsis*, these transporters are localized to the chloroplast envelope and participate in sulfate uptake into chloroplasts, thereby influencing sulfate assimilation and ABA biosynthesis [21,22,23,24]. Notably, Group III *SULTRs* in *O. sativa* and *Arabidopsis* have distinct subcellular localizations and functional profiles, suggesting species-specific adaptations [25]. Group IV contains only two members, *AtSULTR4;1* and *AtSULTR4;2*, both localized to the vacuolar membrane, where they are implicated in remobilizing sulfate from vacuoles to support cellular metabolism [2,23,24].

Chinese cabbage (*B. rapa* L.), as a key economically important vegetable crop in China, exhibits a strong correlation between its yield and sulfur availability. As a vital nutrient element, sulfur participates in protein synthesis and enzyme activation, directly affecting leaf development, photosynthetic efficiency, and stress resistance. Research has shown that when soil available sulfur levels drop below 15 mg/kg, Chinese cabbage manifests typical sulfur deficiency symptoms, including stunted growth and leaf chlorosis, leading to significant biomass reduction. Notably, sulfur deficiency increases the crop’s susceptibility to abiotic stresses including salt and low temperature [26,27]. To date, fundamental knowledge regarding *SULTRs* (sulfate transporter genes) in *B. rapa* has not been systematically characterized. In this study, to explore the evolution of *SULTRs* in this species, we performed genome-wide identification and analyses covering phylogenetic classification, chromosomal distribution, gene structure, conserved protein motifs, and *cis*-regulatory elements [28,29]. Furthermore, we examined *SULTR* expression patterns under salt and low-temperature stress conditions. This work establishes a foundation for elucidating the functional roles of *SULTR* genes in *B. rapa*, offering potential avenues for improving stress tolerance and yield through targeted sulfur management strategies.

## 2. Results

### 2.1. Systematic Identification and Analysis of BrSULTR Gene Family

To screen for candidate *SULTR* genes in *B. rapa*, genome-wide similarity searches were conducted using the amino acid sequences of *A. thaliana SULTR* genes as reference queries. This research identified 19 *SULTR* homologs, designated *BrSULTR1* to *BrSULTR19*, which were mapped unevenly across seven chromosomes in the *B. rapa* genome (Table 1). The encoded proteins exhibited lengths varying from 573 amino acids (*BrSULTR19*) to 695 amino acids (*BrSULTR18*), with corresponding calculated molecular masses ranging from 62.91 kDa to 75.93 kDa. The isoelectric point (pI) spanned from 7.15 (*BrSULTR19*) to 9.62 (*BrSULTR16*). Subcellular localization predictions indicated that all BrSULTR proteins are localized in the plasma membrane. Analysis with the TMHMM server further revealed that each BrSULTR protein contains 8–12 predicted transmembrane domains, suggesting strong sulfate transport potential and the ability to respond rapidly to external signals (Figure S1A–D). Together, these findings collectively elucidate the structural and functional features of the *BrSULTR* gene family.

### 2.2. Evolutionary Analysis and Subgroup Classification of the BrSULTR Family

**Figure 1:**
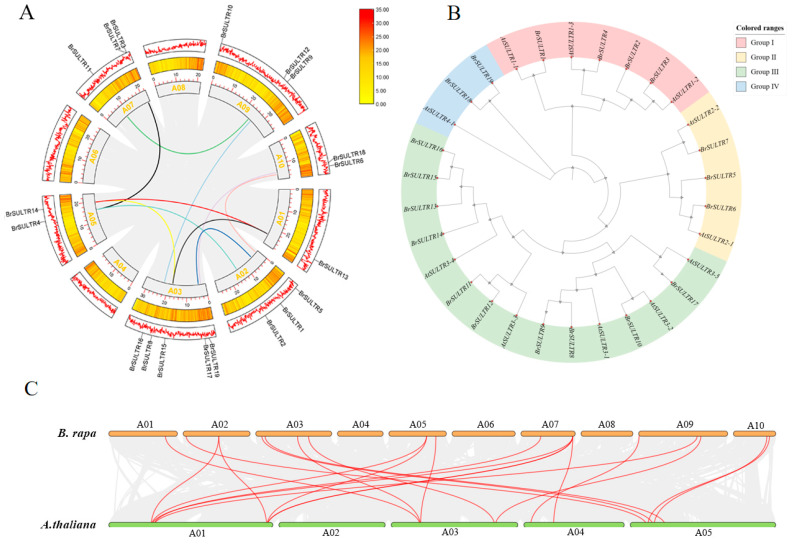
(**A**) Chromosomal distribution and replication events of *BrSULTR* genes in *B. rapa*. Chromosomes are depicted as outermost segments, with gene density displayed in intermediate and inner tracks. Color-coded arcs in the mid-region represent collinear relationships among *BrSULTR* genes. (**B**) Phylogenetic tree of *SULTR* in *B. rapa* and *A. thaliana*. Branches are colored by subgroup: purple for Group I, yellow for Group II, green for Group III, and blue for Group IV. (**C**) Syntenic analysis of *SULTR* between *B. rapa* and *A. thaliana*. Red lines connect homologous genes.

Based on the genomic sequences of the *BrSULTR* genes, we systematically examined their chromosomal arrangement and homology relationships. All 19 *BrSULTR* genes were localized to seven chromosomes of *B. rapa*, with none identified on chromosomes 04, 06, or 08 (Chr04, Chr06, Chr08), indicating a non-uniform distribution pattern. The genes were scattered across the chromosomes, with the number of genes per chromosome varying from one to five. Notably, chromosome 01 (Chr01) harbored five genes, the highest number observed, while chromosome 10 (Chr10) contained only one member of the *BrSULTR* family (Figure 1A). Evolutionary analysis further identified 10 segmental duplication events among the 19 *BrSULTR* genes (Figure 1A). These findings demonstrate that the *BrSULTR* gene family is distributed in a highly uneven manner in the *B. rapa* genome.

To elucidate the evolutionary connections within the *BrSULTR* gene family, a phylogenetic tree was generated based on the aligned SULTR protein sequences from *A. thaliana* and *B. rapa*. The tree topology resolved four distinct clades, designated Group I, Group II, Group III, and Group IV, which reflect their evolutionary relationships (Figure 1B). Among the 19 identified *BrSULTR* genes, Group I contained four *BrSULTR* members. Group II contained three *BrSULTR* members. Group III contained the most populous group, with 10 *BrSULTR* members, accounting for 52.6% of the total. In contrast, Group IV contained two *BrSULTR* members. The results imply that there is homology between SULTR proteins within the same species, suggesting potential functional similarities.

To assess the evolutionary conservation of *SULTR* genes, synteny relationships were conducted between *B. rapa* and *A. thaliana*. Nineteen *BrSULTR* genes were found to be syntenic with their orthologs in *A. thaliana*, reflecting a strong evolutionary relationship between the two species. These findings collectively imply that the *BrSULTR* genes may have potential functional similarities (Figure 1C).

### 2.3. Evolutionary Relationships, Gene Structure, and Conserved Motifs of BrSULTR Family

**Figure 2:**
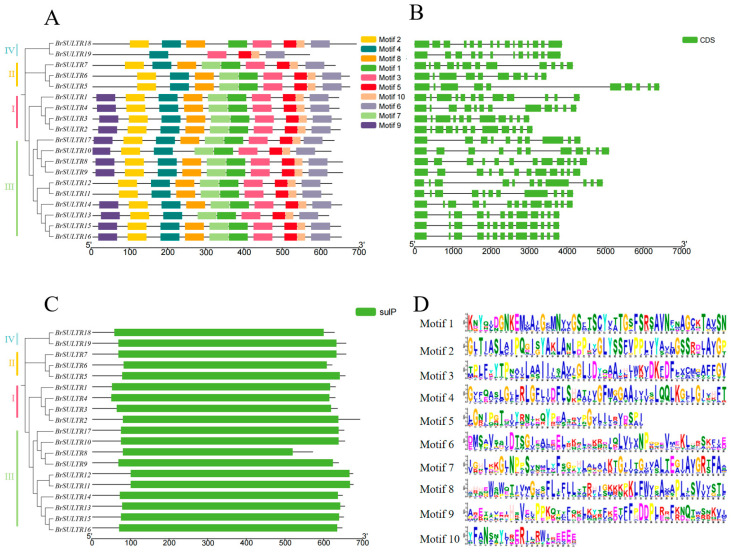
Structural and conserved motif analysis of *BrSULTR* genes. (**A**) Conserved motifs identified in BrSULTR protein. (**B**) Exon–intron organization of *BrSULTR* genes. Green boxes for exons, and lines for introns. (**C**) The sulfate transporter domain regions of *BrSULTRs*. (**D**) Sequence conservation of *SULTR* motifs. The total height of each stack reflects the conservation level at the corresponding site, with letter height representing the frequency of each amino acid. The different colors of letters represent different amino acids.

To systematically investigate the structural architecture of *BrSULTR* genes, we examined the exon–intron architecture and conserved motifs across the 19 *BrSULTR* genes. We revealed that each gene harbors 5 to 10 motifs, with the fewest (5 motifs) observed in *BrSULTR19*, which belongs to Group IV. All conserved motifs were conserved in Group I and Group III, except for *BrSULTR11* and *BrSULTR12*. Meanwhile, *BrSULTR5*, *BrSULTR6*, and *BrSULTR7* contained nine motifs each in Group II, lacking only Motif 9 (Figure 2A,D and Table 2). Moreover, each *BrSULTR* gene contained ten or more exons (Figure 2B). These findings further indicate that *BrSULTR* genes share highly similar structural domains and conserved functions.

Notably, all 19 BrSULTR proteins were found to contain a characteristic sulfate transporter (sulP) domain in their N-terminus (Figure 2C). Previous research showed that the sulP domain is typically localized to the chloroplast envelope, where it supports sulfate uptake and assimilation within chloroplasts [30,31]. These findings suggest that the putative BrSULTR proteins have a similar transmembrane transport function.

### 2.4. Secondary and Tertiary Structural Analysis of BrSULTR Proteins

**Figure 3:**
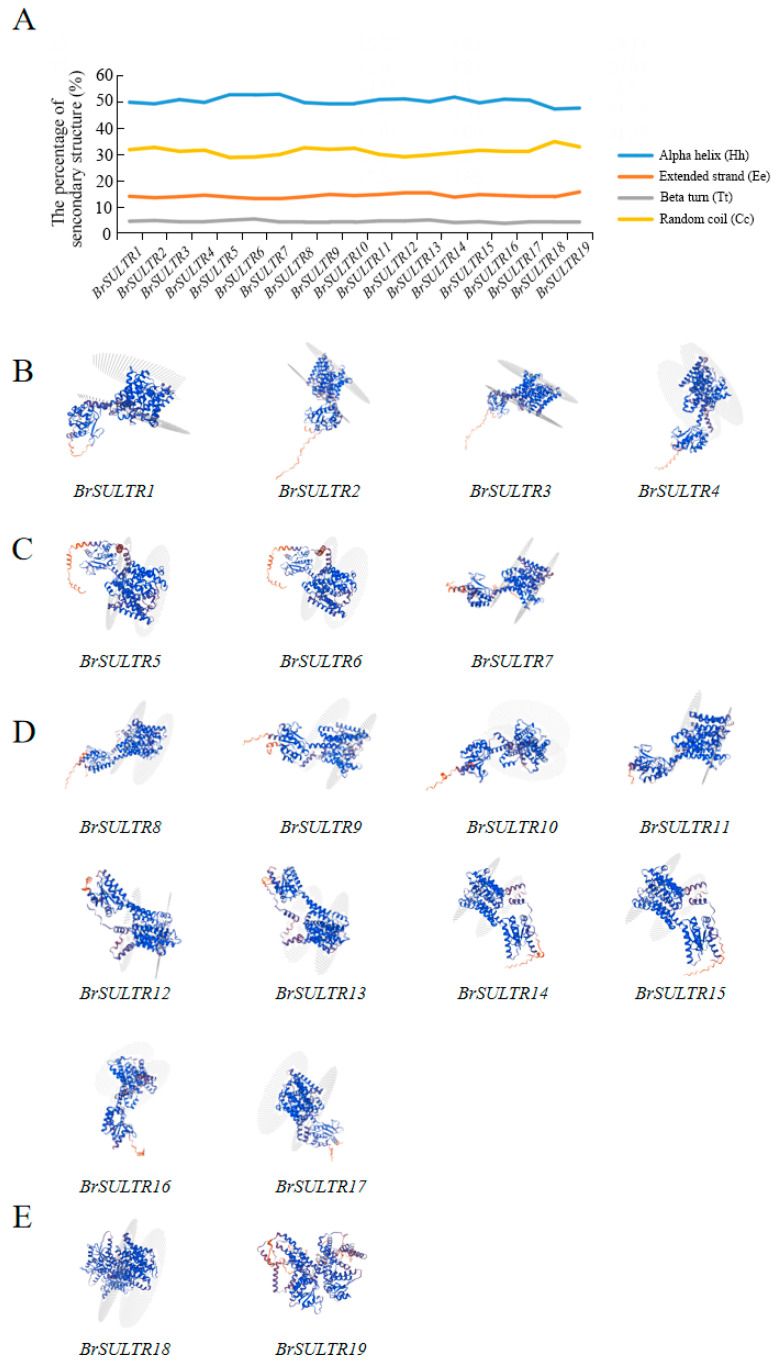
This model is based on idealized conditions for static conformation prediction. (**A**) Secondary architecture of SULTR proteins in *B. rapa*. (**B**–**E**) Tertiary structure of SULTRs from different groups in *B. rapa*: (**B**) Group I, (**C**) Group II, (**D**) Group III and (**E**) Group IV. The tertiary structures are displayed as ribbon diagrams, with a color gradient from red (N-terminus) to blue (C-terminus).

The biological function of a protein is intimately connected to its structural configuration. Hence, investigating the structure of SULTR proteins can shed light on their structural characteristics and evolutionary relationships. Secondary structure analysis of SULTR proteins in *B. rapa* demonstrated that they consisted of *α* helices (Hh), extended strands (Ee), *β* turns (Tt), and random coils (Cc), with these components maintaining a relatively stable ratio (Figure 3A). *α* helices accounted for the largest proportion of BrSULTR secondary structures, which may be related to the functional role of transmembrane proteins. Each SULTR protein in different subgroups has multiple transmembrane domains (Figure S1), all of which are essential for the activity and stability of SULTR proteins. Building on secondary structure predictions, the tertiary structure of SULTR proteins exhibited high structural similarity in different subgroups, which further indicates that homologous structures were maintained during evolution, facilitating the transmembrane transport of sulfur (Figure 3B–E).

### 2.5. Cis-Regulatory Element Analysis of BrSULTRs

**Figure 4:**
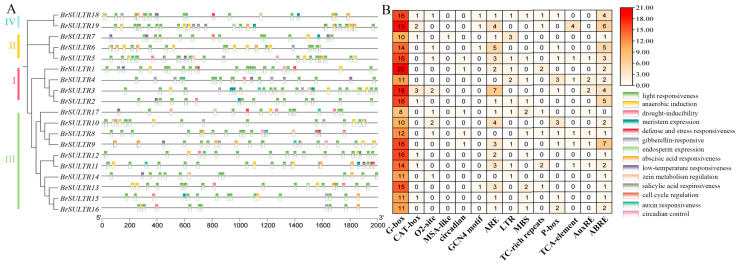
Analysis of *cis*-regulatory elements in the promoter regions of *SULTR* genes in *B. rapa*. (**A**) The varied colors and corresponding numbers in *BrSULTR* gene promoters reflect the diversity of their *cis*-regulatory elements. (**B**) Different *cis*-regulatory elements and their relative positions in each *BrSULTR* gene are shown by colored blocks.

To gain further insights into the potential biological roles of *BrSULTR* family members, we examined *cis*-regulatory elements located within the 2000 bp region upstream of the transcriptional start site (ATG) using the PlantCare database. The identified *cis*-regulatory elements were grouped into three main categories: (1) plant growth-related elements, which are involved in meristem or endosperm development, light responsiveness, and cell cycle regulation; (2) hormone-responsive elements, such as those for auxin, gibberellin, abscisic acid, and salicylic acid; and (3) stress-responsive elements, covering motifs associated with anaerobic induction, drought responsiveness, and low-temperature tolerance (Figure 4). Among the analyzed genes, *BrSULTR5* and *BrSULTR18* harbor the greatest number of *cis*-regulatory elements, predominantly connected to phytohormone signaling, stress adaptation and developmental regulation. In contrast, *BrSULTR14* contains the fewest elements, most of which are development-related. Notably, all *BrSULTR* family members contained abundant light-responsive elements (G-box), suggesting an important role for sulfate transporters in chlorophyll biosynthesis and photosynthetic enzyme activity. Furthermore, approximately 84.2% of these genes contained anaerobic induction elements, and 52.6% contained elements conferring low-temperature responsiveness and drought inducibility, suggesting that *BrSULTR* genes are likely to participate in plant development and growth, and contribute to adaptive responses under abiotic stress conditions, which suggests their regulation by a variety of physiological and environmental signals.

### 2.6. BrSULTR Gene Expression Analysis

#### 2.6.1. Tissue-Specific Expression Patterns of *SULTR* Family Genes

**Figure 5:**
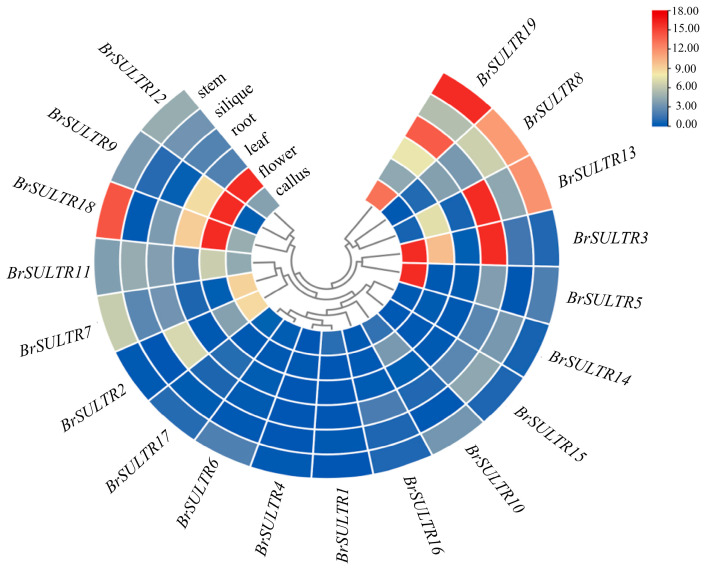
The expression profiles of *BrSULTR* genes in distinct tissues. Deeper red hues indicate higher levels, while cooler tones represent lower levels.

A systematic investigation of *BrSULTR* gene expression in diverse tissues revealed distinct expression profiles for each member of the *BrSULTR* family. The 19 *BrSULTR* genes showed expression in six tissue types, including root, stem, leaf, flower, silique, and callus (Figure 5, Table S2). Tissue-specific expression analysis showed distinct and varied patterns across *BrSULTR* family genes. *BrSULTR8*, *BrSULTR11*, *BrSULTR13*, and *BrSULTR19* were highly expressed in stems. *BrSULTR3*, *BrSULTR13* and *BrSULTR19* were highly expressed in roots. *BrSULTR9*, *BrSULTR11*, and *BrSULTR12* were predominantly expressed in flowers. *BrSULTR3* and *BrSULTR5* were predominantly expressed in callus. The above results imply that the expression patterns of *BrSULTR* genes are likely regulated by multiple processes, such as nutrient transport, stress response, developmental regulation, and many other complex biological pathways.

#### 2.6.2. Analysis of *BrSULTR* Genes in Responses to Abiotic Stress

To further explore the involvement of the *BrSULTR* gene family in response to abiotic stress, we utilized transcriptome and proteome assays, along with qRT-PCR analysis (Table S1), to examine changes in *BrSULTR* expression under salt and low-temperature stress treatments. The expression profiles of *BrSULTRs* were consistent across transcriptome, proteome, and qRT-PCR analyses, showing upregulation or downregulation within the same treatment time point.

**Figure 6:**
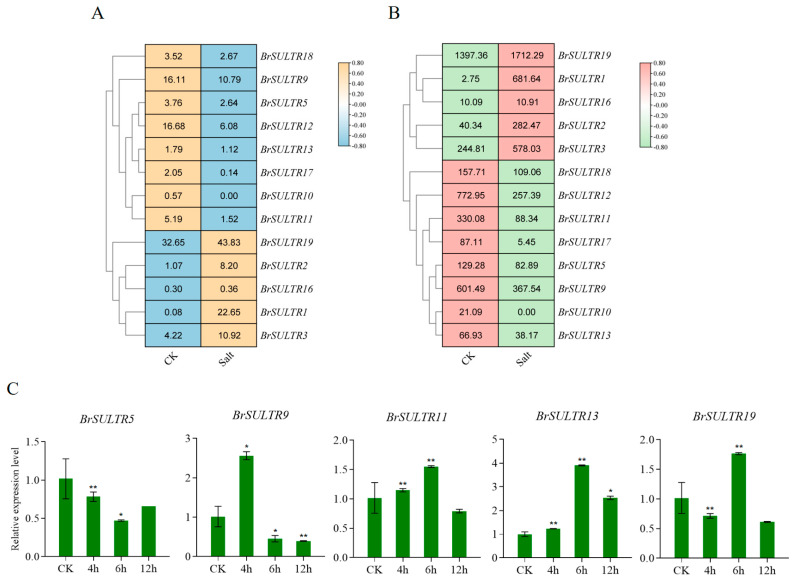
Expression levels of *BrSULTR* genes under salt stress. (**A**) Transcript abundance of *BrSULTRs* following treatment with 150 mM NaCl, as assessed by RNA-seq. (**B**) Protein abundance of BrSULTRs under 150 mM NaCl treatment, detected via proteomic analysis. (**C**) Relative transcript levels of *BrSULTR* genes under 150 mM NaCl treatment were analyzed using qRT-PCR. Data are presented as means; errors are shown as ±SD (*t*-test, * *p* < 0.05, ** *p* < 0.01).

Under salt stress, the expression of *BrSULTR* genes was markedly altered. The results showed that five *BrSULTR* genes (*BrSULTR1*, *BrSULTR2*, *BrSULTR3*, *BrSULTR16*, and *BrSULTR19*) were upregulated, while eight *BrSULTR* genes (*BrSULTR5*, *BrSULTR9*, *BrSULTR10*, *BrSULTR11*, *BrSULTR12*, *BrSULTR13*, *BrSULTR17*, and *BrSULTR18*) were downregulated (Figure 6A,B). For qRT-PCR analysis, five *BrSULTR* genes (*BrSULTR5*, *BrSULTR9*, *BrSULTR11*, *BrSULTR13* and *BrSULTR19*) were selected. The results showed that the relative expression of *BrSULTR5* and *BrSULTR9* was downregulated at 6 h after salt treatment, whereas the relative expression of *BrSULTR11*, *BrSULTR13* and *BrSULTR19* was upregulated, peaking at 6 h post-treatment before declining thereafter compared with the control. Notably, the relative expression of *BrSULTR13* was approximately 2-fold higher than that of the control after 12 h treatment (Figure 6C, Tables S3–S5).

**Figure 7:**
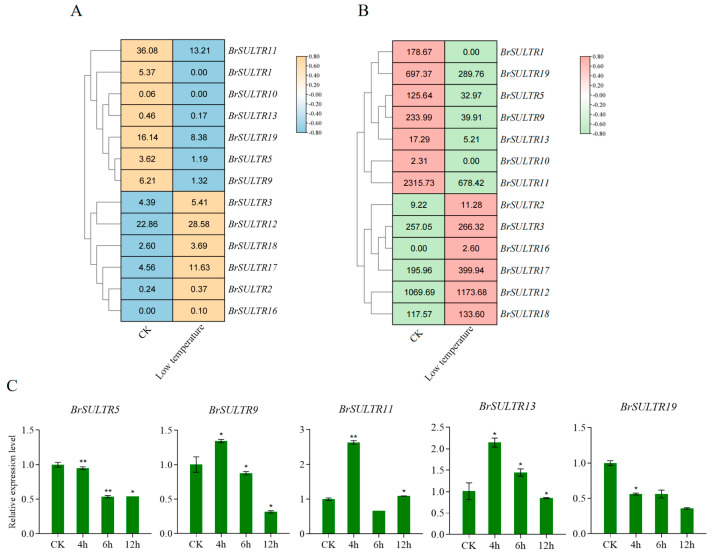
Expression levels of *BrSULTR* genes under low-temperature stress. (**A**) Transcript abundance of BrSULTRs following treatment with 4 °C, as assessed by RNA-seq. (**B**) Protein abundance of BrSULTRs under 4 °C treatment, detected via proteomic analysis. (**C**) Relative transcript levels of *BrSULTR* genes under 4 °C treatment were analyzed using qRT-PCR. Data are presented as means; errors are shown as ±SD (*t*-test, * *p* < 0.05, ** *p* < 0.01).

Low-temperature stress also significantly affected the expression patterns of *BrSULTRs*. The results showed that seven *BrSULTR* genes exhibited decreased expression levels; among them, *BrSULTR11* and *BrSULTR13* were reduced by more than 5-fold relative to the control. *BrSULTR9* was reduced by more than 4-fold and *BrSULTR5* by 3-fold under low-temperature stress (Figure 7A,B). In contrast, the expression level of *BrSULTR17* was increased by more than 2-fold. In addition, the qRT-PCR analysis exhibited that the expression levels of *BrSULTR9*, *BrSULTR11* and *BrSULTR13* initially increased then declined, reaching the lowest levels after 12 h of treatment. Additionally, the expression levels of *BrSULTR5* and *BrSULTR19* showed a gradually decreasing trend under low-temperature stress. These results indicate that *BrSULTR* genes are related to the regulation of responses to both salt and low-temperature stresses (Figure 7C, Tables S3–S5).

### 2.7. Protein–Protein Interaction (PPI) Network Prediction Analysis of SULTR

**Figure 8:**
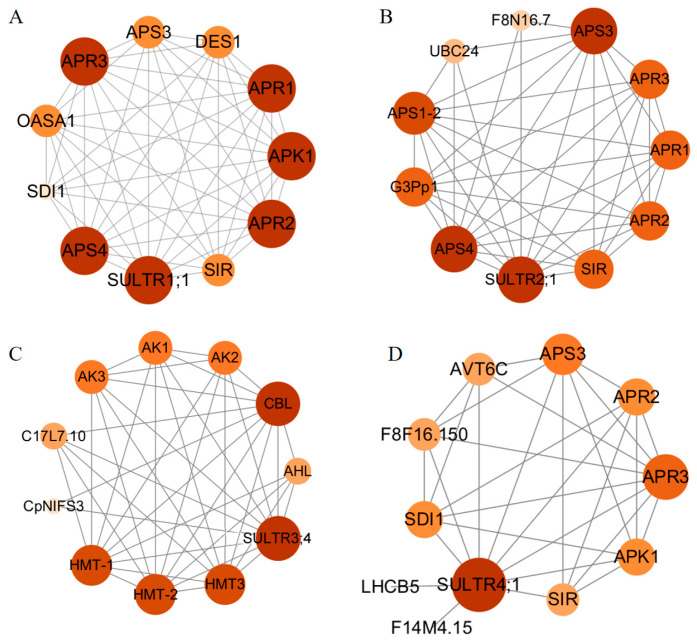
Protein–protein interaction (PPI) networks of four homologous *SULTR* genes in *A. thaliana*: (**A**) Predictive interaction network of AtSULTR1;1. (**B**) Predictive interaction network of AtSULTR2;1. (**C**) Predictive interaction networks of AtSULTR3;4. (**D**) Predictive interaction network of AtSULTR4;1. In each network diagram, the nodes represent proteins, dark orange nodes represent proteins related to abiotic stress, light orange nodes indicate other interacting proteins, and connecting lines indicate predicted associations. The minimum participation score for analysis is 0.150, and remaining parameters were kept at default settings.

Proteins interact with other proteins (protein–protein interactions, PPIs) in their cellular environment to carry out biological processes essential for various cellular behaviors and functions [32,33]. To further explore the *SULTR* gene functions and physical interactions, the interactions of four *A. thaliana* homologous genes with diverse proteins were analyzed using the STRING tool, including AtSULTR1;1 (homologs: BrSULTR1, Figure 8A), AtSULTR2;1 (homologs: BrSULTR5 and BrSULTR6, Figure 8B), AtSULTR3;4 (homologs: BrSULTR13, BrSULTR14, BrSULTR15, and BrSULTR16, Figure 8C), and AtSULTR4;1 (homologs: BrSULTR18 and BrSULTR19, Figure 8D).

It is noteworthy that AtSULTR1;1, AtSULTR1;2, and AtSULTR4;1 proteins all interacted with ATP sulfurylase (APS), 5′-adenylylsulfate reductase (APR), adenylyl-sulfate kinase (APK), and sulfate reductase (SIR), which participate in the biosynthesis of sulfur-containing amino acids, thereby directly influencing the production of proteins and antioxidants and alleviating oxidative stress. Notably, members of the *APR* gene family are significantly upregulated under heavy metal (e.g., cadmium and lead), enhancing plant tolerance to heavy metals by regulating sulfur metabolism. Its promoter region is enriched with stress response elements and directly participates in stress signal transduction. In addition, APK catalyzes the phosphorylation of APS to generate 3′-adenosine-5′-phosphosulfuric acid (PAPS), which is essential for plant disease resistance and heavy metal chelation. SIR catalyzes the reduction of sulfite to sulfide, which serves as a critical step in cysteine biosynthesis. Cysteine, as a precursor of glutathione (GSH) and metallothioneins, plays a pivotal role in stress responses. Under adverse conditions, the upregulation of this metabolic pathway enhances plant antioxidant capacity by promoting the synthesis of these key defense molecules. Homocysteine S-methyltransferase (HMT) physically interacts with AtSULTR3;4, and under salt stress, its gene expression is significantly upregulated, thereby promoting methionine biosynthesis through enhanced metabolic flux [34]. Notably, the accumulated methionine further improves plant salt tolerance by activating abscisic acid (ABA) biosynthesis and signaling pathways, ultimately leading to increased expression of salt-responsive genes involved in stress adaptation. CBL was predicted to interact with AtSULTR3;4. Under salt stress, CBL4 forms a complex with CIPK24, which phosphorylates and activates the SOS1 transporter to promote extracellular Na^+^ efflux; meanwhile, CBL8 enhances cold tolerance by regulating the sugar transporter protein STP1. The above results indicate that the *BrSULTRs* are functionally involved in stress response.

### 2.8. Gene Ontology (GO) Analysis of SULTRs in B. rapa

**Figure 9:**
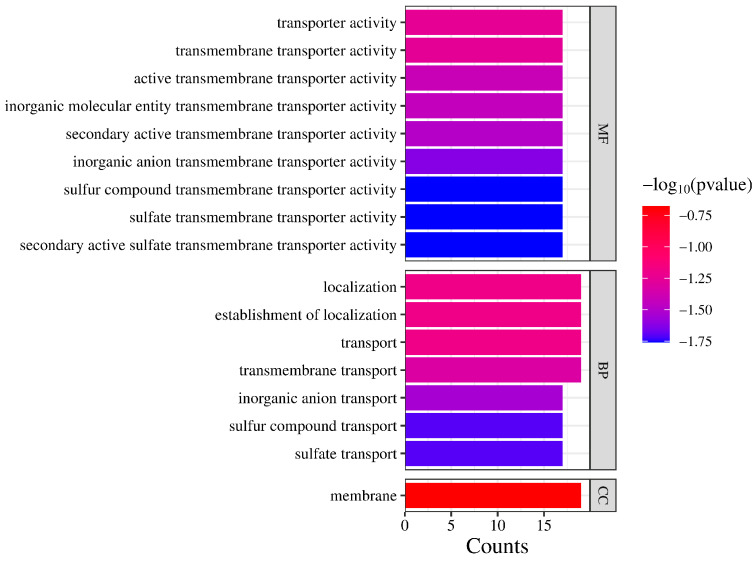
Gene ontology (GO) annotations of *SULTRs* in *B. rapa*. GO terms are categorized into three groups: molecular function (MF), biological process (BP), and cellular component (CC). The y-axis represents individual GO terms, while the x-axis displays the gene count for each term. Bar length represents the −log_10_ (*p*-value).

To better elucidate the function of BrSULTR proteins and the biological processes they are involved in, we conducted a GO analysis to gain insights into their potential functions. *BrSULTR* genes were enriched in GO terms classified into three categories: molecular functions (GO-MFs), biological processes (GO-BPs), and cellular components (GO-CCs) (Figure 9).

In the molecular function section, the chart displays a series of “transporter activity” terms, such as transporter activity, transmembrane transporter activity, and more specific subtypes including inorganic anion transmembrane transporter activity and sulfate transmembrane transporter activity. The biological process section focuses on transport and localization-related terms, including localization, transport, transmembrane transport, and specialized processes such as sulfur compound transport and sulfate transport. Finally, the cellular component section features a single prominent red bar labeled “membrane” (GO:0016020). Together, the GO analysis indicates that *BrSULTRs* are crucial for promoting transmembrane transport of sulfates in plants.

## 3. Discussion

Sulfate, the primary storage form of sulfur nutrition, plays a crucial role in plant sulfur metabolism [35,36]. *SULTR* family members are key players in sulfate uptake, transport, and plant responses to both biotic and abiotic stresses. Nevertheless, the *BrSULTR* gene family has received limited attention in *B. rapa*. In this study, we systematically identified *BrSULTRs* and characterized their physicochemical attributes, structural features, phylogenetic relationships, protein structures, *cis*-regulatory elements, protein–protein interactions, GO enrichment, and expression profiles under salt and low-temperature stresses. These findings enhance our comprehension of *BrSULTR* genes and their functional contributions to plant stress responsiveness.

Compared with *A. thaliana*, the phylogenetic tree of the *BrSULTR* gene family exhibited a high degree of similarity across the four subfamilies, with the conserved SULTR domain present in all 19 *BrSULTR* members. Our analysis identified 10 chromosomal segmental duplications in *BrSULTR* genes, suggesting functional conservation (Figure 2). Furthermore, 20 orthologous gene pairs were identified between *B. rapa* and *A. thaliana*, indicating a high level of genomic homology [37]. This suggests that the functions of *BrSULTR* genes may be inferred from those of *AtSULTRs*. In *A. thaliana*, the STAS domain extends into the cytoplasm and facilitates the proper localization of SULTR proteins within the membrane [9,38,39]. Moreover, conserved domain and motif analyses further clarified relationships among members and their potential functions. Motifs 3, 4, 5, 6, and 10 were found in all *BrSULTR* members, suggesting their critical role in sulfate transport. Our findings also showed that *SULTR* family members in *B. rapa* contain a SulP domain, indicating their role in sulfate uptake and assimilation in chloroplasts [30,31]. Subcellular localization analysis confirmed that all BrSULTR proteins are located on the plasma membrane, enabling the release of stored SO_4_^2−^ into the cytosol [24]. Growing evidence suggests that sulfate uptake, transport, and assimilation contribute to plant tolerance to salt, drought, and heat stress [40]. Accordingly, we examined *BrSULTR* gene expression under salt and low-temperature stress conditions.

The number of plant hormone-responsive *cis*-regulatory elements varied among the *BrSULTR* genes. Promoter analysis identified abscisic acid responsiveness (ABRE) and defense- and stress-responsive elements (TC-rich repeats) within the promoter of *BrSULTR11*, indicating that *BrSULTR* genes may be involved in regulating plant growth, development, and stress responses in *B. rapa*. Sulfate is a vital component of plant sulfur nutrition, as roots and shoots transport SO_4_^2−^ from the soil environment [34,36]. In our study, tissue-specific expression analysis revealed that *BrSULTRs* were predominantly expressed in roots and stems, which supports their proposed role in sulfate source-sink transport. *BrSULTR11* was highly expressed in stems, implying an important function in sulfate long-distance translocation. Water deficit in plants leads to stomatal closure, with abscisic acid (ABA) acting as the key signal that transmits information from roots to shoots [41]. Previous studies showed that ABA is a central hormone in abiotic stress responses, inducing the expression of stress-related genes under conditions such as salinity [42,43,44]. In addition, the promoter region of *BrSULTR11* contained ABRE elements, and RNA-seq and qRT-PCR analysis both confirm its upregulation under salt stress. From these findings, we postulate that *BrSULTR11* may contribute to the adaptation of *B. rapa* to salt stress.

In plant-environment interactions, external conditions can affect the morphological structure and physicochemical characteristics of plants, while plants can also adapt to the environment. In this study, each *BrSULTR* gene contained a varying number of plant hormone *cis*-acting elements. *BrSULTR9* was found to contain the highest number of ABRE elements, along with P-box (gibberellin-responsive) elements. Gibberellin (GA) regulates responses to external stress by activating the DELLA protein, a repressor of the GA signaling pathway, to repress protein degradation [45]. This mechanism can suppress plant development while enhancing stress tolerance by reducing ROS accumulation [46]. Plant growth and development are regulated by multiple hormones. Plants respond to various stresses, including those associated with abscisic acid, gibberellin, and salicylic acid, which in turn modulate different aspects of their growth and development. According to RNA-seq data, the expression levels of most *BrSULTRs*, including *BrSULTR9*, exhibited downregulation in response to salt and low-temperature stress. Additionally, qRT-PCR data showed that the expression of *BrSULTR9* decreased threefold following 6 h of salt stress treatment, as well as after 12 h of low-temperature stress treatment. These results illustrate that *BrSULTR9* may be actively downregulated in response to salt stress and low-temperature stress.

Secondary structure analysis indicated that alpha helices had the highest proportion across all BrSULTR proteins, while the proportion of beta turns was the lowest. In the comparison of members within the same subgroup, the tertiary structure and transmembrane architectures arrangement exhibit a high level of structural conservation, reflecting the stable maintenance of homologous conformation during evolution, which indicates that this type of protein has a conserved evolutionary origin in its basic functional structural framework. However, under abiotic stress conditions, its conformation may undergo dynamic changes accompanied by various physiological adaptive adjustments, which play an important role in maintaining cellular homeostasis or sensing environmental changes. Moreover, *BrSULTR* genes were prominently enriched in Gene Ontology (GO) terms associated with transmembrane transporter activity. Prediction of SULTR protein transmembrane structure in *B. rapa* revealed that BrSULTR5 has 12 transmembrane domains, suggesting that it may respond more rapidly to external stimuli. This provides foundational information for future studies on BrSULTR protein functions. PPI predictions indicated that AtSULTR2;1, a homolog of BrSULTR5, interacts with APS, which plants use for assimilatory sulfate reduction. Previous studies have shown that sulfate is first activated by ATP sulfurylase to form adenosine 5′-phosphosulfate (APS), and APS reductase (APR) plays a key role in regulating sulfur assimilation and plant responses to multiple environmental stresses [47,48]. At the final stage of sulfur assimilation, sulfur-containing compounds such as GSH are produced, enhancing plant tolerance to abiotic stresses. Thus, ATP-S contributes to plant stress tolerance through S-compound-mediated mechanisms [49]. Transcriptomic and proteomic analyses showed that *BrSULTR5* was downregulated more than threefold under low-temperature stress. qRT-PCR analysis further revealed a 2-fold downregulation after 6 h treatment, which underscores a pivotal role for *BrSULTR5* in the low-temperature stress response of *B. rapa*.

## 4. Conclusions

This study performed a systematic genomic identification and functional characterization of the sulfate transporter gene family members in *B. rapa*. The analysis of phylogenetic classification, gene structure, conserved motif dissection, *cis*-regulatory element prediction, and expression profiles further links sulfur metabolism with stress response. These findings offer a foundation for subsequent functional investigations of BrSULTRs and enhance the understanding of sulfate transport in *B. rapa*.

## 5. Materials and Methods

### 5.1. Identification of BrSULTR Family Members and Subcellular Localization Analysis

To screen for *BrSULTR* genes in the *B. rapa* genome, complete SULTR protein sequences from *A. thaliana* were retrieved from the TAIR database (https://www.arabidopsis.org/) (accessed on 12 November 2024). Putative *BrSULTRs* were subsequently identified by performing BLASTP searches against the *B. rapa* genome using *A. thaliana* SULTR protein sequences as queries, followed by analysis of conserved structural domains. Pfam (http://pfam.xfam.org/) (accessed on 12 November 2024) and SMART 9.0 databases (http://smart.embl-heidelberg.de/) (accessed on 12 November 2024) were used to perform genome-wide annotation of protein domains in *B. rapa* [50,51].

The physicochemical attributes of the *BrSULTR* family were analyzed with Expasy (http://web.expasy.org/) (accessed on 16 November 2024). Subcellular localization and transmembrane structures of SULTR family proteins were predicted using default parameters in WoLF PSORT (v0.2) (http://wolfpsort.org/) (accessed on 16 November 2024) [50,52].

### 5.2. Chromosomal Localization, Synteny, and Evolutionary Phylogenetic Analysis

Chromosomal localization of *BrSULTR* genes was mapped with TBtools, and synteny relationships with *A. thaliana SULTR* genes were analyzed using MCScanX (v1.0.0) [53,54]. A maximum likelihood phylogenetic tree was constructed in MEGA X with the proximity method, bootstrap support of 1000 replicates, and remaining parameters set to default [55]. The resulting phylogenetic tree was visualized and annotated using iTOL (https://itol.embl.de/) (accessed on 16 November 2024) [56].

### 5.3. Analysis of Conserved Motifs, Gene Structure, and Functional Domains

Intron–exon structures were analyzed via GSDS (http://gsds.gao-lab.org/) (accessed on 22 November 2024) [57]. Conserved motifs were detected using Pfam and MEME suite [58], with parameters set to a maximum of 10 motifs, each allowed to appear zero or one time per sequence. Conserved domains were detected via NCBI’s Batch CD-Search (https://www.ncbi.nlm.nih.gov) (accessed on 22 November 2024) and visualized with “Visualize Pfam Domain Pattern” (from Pfam Search) in TBtools [59].

### 5.4. Protein Secondary Structure, Tertiary Structure and Transmembrane Domains Analysis

Secondary structures of BrSULTR proteins were predicted using the PRABI (https://prabi.ibcp.fr/htm/site/web/app.php/home) (accessed on 22 November 2024). Tertiary structure models were generated via the SWISS-MODEL platform ((https://swissmodel.expasy.org/) (accessed on 22 November 2024) [60]. Transmembrane domains were predicted with the TMHMM Server v2.0 (https://services.healthtech.dtu.dk/service.php?TMHMM-2.0) (accessed on 22 November 2024) [61].

### 5.5. Cis-Regulatory Analysis

To investigate promoter function, a 2 kb region upstream of the translation start site for each *BrSULTR* gene was extracted. *Cis*-regulatory elements were then systematically identified and analyzed using the PlantCARE database with default parameters [62].

### 5.6. Tissue-Specific Expression Profiling

To examine the expression patterns of *BrSULTR* across different tissues, expression levels were retrieved from the Brassicaceae Database (http://brassicadb.cn/) (accessed on 03 December 2024) for roots, stems, leaves, flowers, and siliques. Tissue-specific expression patterns were visualized and analyzed with TBtools (v1.120).

### 5.7. Stress Treatments and Integrated Transcriptome, Proteome, and qRT-PCR Analysis

Seeds of the cultivar ‘chiifu’ were germinated and grown on a modified MS medium (supplemented with vitamins, sucrose, and agar) (PM10121-307) under controlled conditions (24 °C, relative humidity: 66%, 16 h light/8 h dark). Salt stress (150 mM NaCl) and low-temperature stress (4 °C) treatments were applied for 0, 4, 6, and 12 h at the College of Agriculture, Hengxing University of Science and Technology (Qingdao, Shandong, China). Entire leaf samples were collected at designated time points, snap-frozen in liquid nitrogen, and subsequently processed for RNA extraction and qRT-PCR analysis. Three independent biological replicates were prepared for each treatment group. Seedlings treated with 6 h of salt stress and low-temperature stress were used for transcriptome and proteome analysis.

We used the FastPure^®^ Cell/Tissue Total RNA Isolation Kit V2 (Vazyme Biotech Co., Ltd., Nanjing, China) to extract total RNA, and RNA quality was assessed by 1% agarose gel electrophoresis. Concentration and purity were determined using a Thermo NanoDrop One spectrophotometer. First-strand cDNA was synthesized via HiScript^®^ II Q RT SuperMix, diluted tenfold, and stored at −20 °C. The qRT-PCR reactions were performed in 20 μL volumes containing 10.0 μL 2 × SYBR qPCR Master Mix (Vazyme Biotech Co., Ltd., Nanjing, China), 0.4 μL each of forward and reverse primers (10 μM), 1.0 μL cDNA template, and 8.2 μL nuclease-free water. The thermal cycling program was: 95 °C for 30 s; 40 cycles of 95 °C for 10 s; 60 °C for 20 s; followed by a melt-curve stage: 95 °C for 24 s; 60 °C for 60 s; and 95 °C for 7 s. *BraActin2* was used as the reference gene, and relative expression levels were calculated using the 2^−∆∆CT^ method [63]. Primer sequences are provided in Table S1.

### 5.8. Protein–Protein Interaction (PPI) Network Analysis

PPI networks were constructed using the STRING (https://cn.string-db.org/) (accessed on 14 December 2024) database with default settings. The resulting interaction data were visualized and analyzed in Cytoscape (version 3.9.1) [64,65].

### 5.9. Gene Ontology (GO) Enrichment Analysis

Gene Ontology (GO) is a widely adopted standardized framework for annotating gene and gene product functions. Enrichment analysis of candidate genes was performed using the online platform g: Profiler (https://biit.cs.ut.ee/gprofiler/gost/) (accessed on 15 December 2024) [66].

## Figures and Tables

**Table 1:** Characteristics of *BrSULTRs*.

Gene ID	Gene Name	Gene Start Position	Gene End Position	Chr. No	Isoelectric Point	CDS (bp)	Amino Acid (aa)	MW (kDa)	*A. thaliana* ID	*A. thaliana* Name	Subcellular Localization	
*Bra022623*	*BrSULTR1*	8,452,913	8,457,244	A02	9.12	1950	649	70.51	*AT4G08620*	*AtSULTR1;1*	Plasma membrane	
*Bra008340*	*BrSULTR2*	14,828,660	14,831,752	A02	9.12	1962	653	71.63	*AT1G78000*	*AtSULTR1;2*	Plasma membrane	
*Bra015641*	*BrSULTR3*	21,596,928	21,599,937	A07	8.81	1971	656	71.73	*AT1G78000*	*AtSULTR1;2*	Plasma membrane	
*Bra031368*	*BrSULTR4*	15,693,577	15,697,823	A05	9.24	1953	650	71.46	*AT1G22150*	*AtSULTR1;3*	Plasma membrane	
*Bra028595*	*BrSULTR5*	1,221,842	1,228,264	A02	9.07	2037	678	73.93	*AT5G10180*	*AtSULTR2;1*	Plasma membrane	
*Bra009052*	*BrSULTR6*	15,035,724	15,039,183	A10	8.71	2034	677	74.06	*AT5G10180*	*AtSULTR2;1*	Plasma membrane	
*Bra015642*	*BrSULTR7*	21,584,412	21,588,565	A07	9.13	1923	640	70.23	*AT1G77990*	*AtSULTR2;2*	Plasma membrane	
*Bra012831*	*BrSULTR8*	22,023,178	22,022,702	A03	8.83	1980	659	72.63	*AT3G51895*	*AtSULTR3;1*	Plasma membrane	
*Bra006882*	*BrSULTR9*	25,962,205	25,966,548	A09	8.61	1980	659	72.81	*AT3G51895*	*AtSULTR3;1*	Plasma membrane	
*Bra036249*	*BrSULTR10*	102,488	107,591	A09	8.88	1890	629	68.95	*AT4G02700*	*AtSULTR3;2*	Plasma membrane	
*Bra012359*	*BrSULTR11*	8,231,401	8,235,557	A07	9.34	1899	632	69.24	*AT1G23090*	*AtSULTR3;3*	Plasma membrane	
*Bra024562*	*BrSULTR12*	24,397,751	24,402,689	A09	9.36	1896	631	69.18	*AT1G23090*	*AtSULTR3;3*	Plasma membrane	
*Bra021152*	*BrSULTR13*	23,685,983	23,689,782	A01	9.42	1872	623	67.95	*AT3G15900*	*AtSULTR3;4*	Plasma membrane	
*Bra027197*	*BrSULTR14*	19,625,378	19,629,523	A05	9.51	1974	657	71.95	*AT3G15900*	*AtSULTR3;4*	Plasma membrane	
*Bra001617*	*BrSULTR15*	17,434,242	17,438,037	A03	9.61	1965	654	71.67	*AT3G15900*	*AtSULTR3;4*	Plasma membrane	
*Bra019289*	*BrSULTR16*	25,193,306	25,197,104	A03	9.62	1971	656	71.94	*AT3G15900*	*AtSULTR3;4*	Plasma membrane	
*Bra006511*	*BrSULTR17*	3,849,090	3,853,440	A03	8.6	1914	637	70.54	*AT5G19600*	*AtSULTR3;5*	Plasma membrane	
*Bra008820*	*BrSULTR18*	13,980,237	13,984,107	A10	8.33	2088	695	75.93	*AT5G13550*	*AtSULTR4;1*	Plasma membrane	
*Bra006206*	*BrSULTR19*	2,512,475	2,516,305	A03	7.15	1722	573	62.91	*AT5G13550*	*AtSULTR4;1*	Plasma membrane	
Aveage	\			\	8.97		647.79	71.01	\	\		

**Table 2:** Information on *SULTR* motifs in *B. rapa*.

Motif	Motif Consensus
Motif 1	KNYQIDGNKEMIAIGFMNVVGSFTSCYVTTGSFSRSAVNFNAGCKTAVSN
Motif 2	GLTIASLAIPQGISYAKLANLPPIYGLYSSFVPPLVYAVLGSSRDLAVGP
Motif 3	TPLFYYTPNAILAAIIJSAVJGLIDYQAAIHJWKVDKFDFLVCMGAFFGV
Motif 4	GVFZASLGLLRLGFLIDFLSHATIVGFMGGAAIVISLQQLKGLLGITHFT
Motif 5	LGNIPGTDIYRNIKQYPEATRIPGVLILRVDSPI
Motif 6	DMSAVTNIDTSGIEALEELRKSLEKRDJQLVJANPGGEVMEKLHRSKFIE
Motif 7	IGHLKKGLNPPSVNMLYFSGPHLALAIKTGIITGIVALTEGIAVGRTFAA
Motif 8	HHHEWSWQTIVMGVSFLIFLLTTRHIGKKKPKLFWVPAVAPLJSVIISTL
Motif 9	ARETIVEIHSVELPPKQTTFKKLKYTFKETFFPDDPLRRFKBQTWSKKVI
Motif 10	YFANSNYLRERILRWIREEEE

## Data Availability

Data are contained within the article and Supplementary Materials.

## Supplementary Materials

The following supporting information can be downloaded at: https://www.mdpi.com/article/10.3390/genes17040394/s1. Table S1: List of primers used for qRT-PCR. Table S2: Tissue-specific expression data (TPM value). Table S3: The expression of *BrSULTR* genes after 150 mM NaCl and 4 °C treatments in transcriptome. Table S4: The expression of *BrSULTR* genes after 150 mM NaCl and 4 °C treatments in proteome. Table S5: The qRT-PCR data under 150 mM NaCl and 4 °C treatments after analysis. Figure S1: Prediction of BrrSULTR protein transmembrane domains. The x-axis shows the length of the protein sequence, and the y-axis shows the probability.
