# Supplementary figures and images for: Comprehensive Characterization of BrSULTRs Family and Their Expression Profiles Under Salt and Low-Temperature Stresses
Source: Genes (Basel). 2026 Mar 30;17(4):394. doi: 10.3390/genes17040394 (PMC13115570; doi:10.3390/genes17040394)

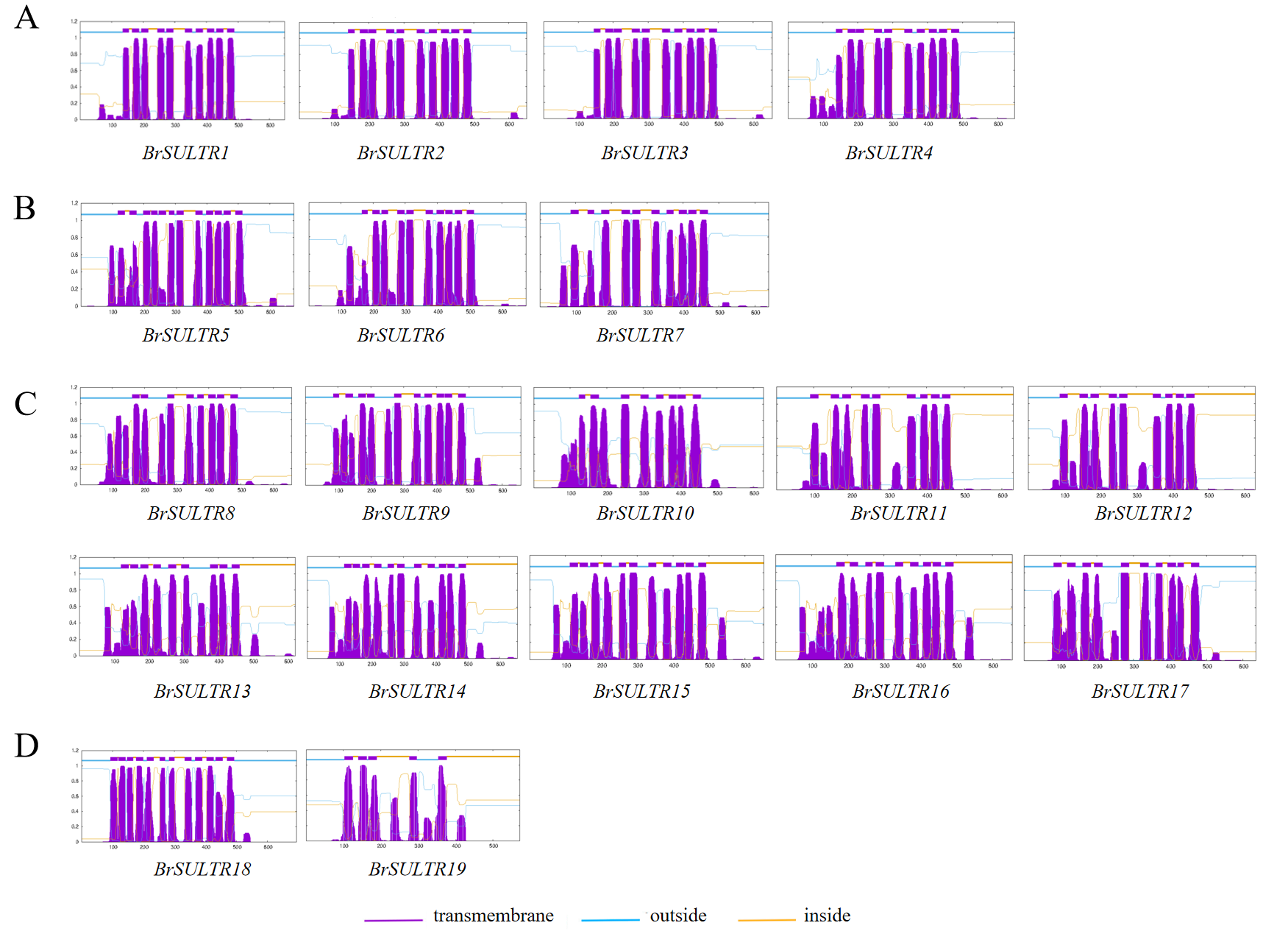

Supplement: Supplementary file 1 [file genes-17-00394-s001.zip › genes-4212472-supplementary/Supplementary materials/figS1.tif]
